# TNF-α–Induced miR-21-3p Promotes Intestinal Barrier Dysfunction by Inhibiting MTDH Expression

**DOI:** 10.3389/fphar.2021.722283

**Published:** 2021-08-12

**Authors:** Zhifeng Jiang, Feiyu Yang, Jingbo Qie, Chaoyuan Jin, Feng Zhang, Jie Shen, Lin Zhang

**Affiliations:** ^1^ Department of Emergency and Critical Care Medicine, Jinshan Hospital, Fudan University, Shanghai, China; ^2^ Department of Emergency and Critical Care Medicine, Jinshan Hospital, Institutes of Biomedical Sciences, Fudan University, Shanghai, China

**Keywords:** TNF-α, miR-21-3p, MTDH, Wnt signaling pathway, intestinal barrier dysfunction

## Abstract

Intestinal barrier dysfunction is characterized by increased intestinal permeability to lumen endotoxin, showing remarkable predisposition to immune enteropathy, and colorectal cancer tumor necrosis factor (TNF)-α is associated with this pathological process, while the mechanism remains unknown. In this study, different doses of TNF-α were used for Caco-2 cell treatment. We discovered that miR-21-3p expression was obviously increased by TNF-α in a dose-dependent manner. Further study demonstrated that TNF-α could upregulate miR-21-3p expression through the NF-κB signaling pathway. Then, TargetScan and miRWalk miRNA–mRNA interaction prediction online tools were introduced, and metadherin (MTDH) was screened out as a potential target of miR-21-3p. We subsequently found that miR-21-3p could directly target the 3′-untranslated region (UTR) of MTDH mRNA and inhibit its expression. Furthermore, it was demonstrated that miR-21-3p could regulate the Wnt signaling pathway by targeting MTDH mRNA, suggesting the effect of miR-21-3p/MTDH/Wnt axis on intestinal barrier dysfunction. Our findings provide a novel potential biomarker and therapeutic target for intestinal barrier dysfunction and related diseases.

## Introduction

As one of the most important barriers of the body, normal intestinal epithelial tissue forms a selectively permeable barrier that regulates the absorption of nutrients and resistance of toxins, antigens, and foreign microorganisms (Turner, 2009; Du et al., 2015). Injury of intestinal epithelial cells is often accompanied by increased permeability of epithelial cells to harmful stimulants, contributing to the occurrence and development of immune enteropathy, and even colorectal cancer (CRC) (Turner, 2009; Odenwald and Turner, 2013; Pang et al., 2021).

The maintenance of intestinal barrier function partly relies on the tight junctions (TJs) between adjacent epithelial cells (Farquhar and Palade, 1963; Li et al., 2021). Dysfunction of the intestinal barrier in patients with inflammatory bowel disease (IBD) is associated with disturbance in the expression or distribution of several TJ proteins, including occludin and claudins (Tsukita et al., 2001; Van Itallie and Anderson, 2006). For example, the decreased number of TJ chains or broken TJ chains obviously occur along with the alteration of the expression levels of occludin and claudins in intestinal tissues from patients with IBD (Hering, Fromm, and Schulzke, 2012). These changes lead to the damage of the intestinal barrier system, resulting in increased permeability of the intestinal wall and the risk of pathogen invasion.

Tumor necrosis factor (TNF)-α, as a proinflammatory cytokine, is a correlative factor that causes enteritis and other inflammatory lesions in patients with celiac disease (CD) (Dolinger et al., 2020; Targownik et al., 2020). Multiple studies have shown the overexpression of intestinal TNF-α in patients with CD (Braegger et al., 1992; Murch et al., 1993; Reibetanz and Germer, 2015; Paredes et al., 2019), and anti-TNF-α antibody therapy has been widely used to treat the patients with IBD (Ricart et al., 2000; Reibetanz and Germer, 2015). Anti-TNF-α antibody drugs also showed good efficacy in several intestinal inflammation animal models (Neurath et al., 1997). It is demonstrated that the high level of TNF-α results in the activation of immunoregulatory pathways by increasing intestinal epithelial permeability (Ma et al., 2004). Moreover, anti-TNF-α antibodies can reestablish intestinal barrier function in patients with CD, suggesting that intestinal barrier damage in patients with IBD is at least partially caused by TNF-α activity. Although the mechanism by which TNF-α affects TJ permeability remains unclear, we previously demonstrated that miR-21-3p was upregulated to increase the intestinal epithelial TJ permeability in the intestinal barrier dysfunction cell model (Zhang et al., 2015; Zhang et al., 2018). These findings indicate that miR-21-3p acts as a mediator linking TNF-α and intestinal barrier dysfunction, which promote us to explore the mechanism about miR-21-3p in intestinal barrier dysfunction.

Herein, miR-21-3p expression was identified to be regulated by the TNF-α/NF-κB signaling pathway, resulting in the downregulation of metadherin (MTDH) expression, which was a key mediator of the Wnt signaling pathway. These findings suggest that the miR-21-3p/MTDH/Wnt axis participates in intestinal barrier dysfunction and provide a better understanding of the pathological process of intestinal barrier dysfunction.

## Materials and Methods

### Tissue Samples

Ten patients with colorectal cancer (CRC) in Jinshan Hospital were selected, and CRC tissue samples were obtained surgically. This study was approved by the Ethics Committee of the Jinshan Hospital.

### Cell Culture

The Caco-2 cell line was obtained from the Chinese Academy of Sciences (CAS, Shanghai, China) and cultured in DMEM with 10% FBS (Gibco, NY, United States) at 37°C.

### Cell Treatment and Transfection

Caco-2 cells were treated with TNF-α (Sigma, United States) at final concentrations of 0, 50, 100, and 150 ng/ml, respectively. In some experiments, Caco-2 cells were stimulated with parthenolide (p65 inhibitor, purchased from TOCRIS) at a final concentration of 5 μmol/L.

Caco-2 cells were transfected with miRNAs or plasmid using Lipofectamine 2000 reagent (Invitrogen). The miR-21-3p mimic (HmiR-SN0315), miR control (CmiR-SN0001-SN), miR-21-3p inhibitor (HmiR-AN0315-SN-10), and miR inhibitor control (CmiR-AN0001-SN) were purchased from FulenGen (Guangzhou, China). The sequence of siRNA that specifically targeted MTDH was chemically synthesized by Sangon (Shanghai, China) (forward: GCA​AUU​CCU​UGG​AUC​UUA​UTT, reverse: AUA​AGA​UCC​AAG​GAA​UUG​CTT).

### Cell Morphology and Immunofluorescence Staining

Caco-2 cells were stimulated with TNF-α, and cell morphology was examined at 24 and 48 h using an optical microscope, respectively. For the immunofluorescence array, the staining of Caco-2 cells was performed using the antibodies against occludin and claudin-1 (Sino Biological, China). Nuclei were visualized by incubation with DAPI. Images were photographed with a Leica fluorescence microscope (DM4000 B, Germany).

### Quantitative Real-Time PCR

TRIzol reagent (Ambion, United States) was used to extract total RNA. The primers against miR-21-3p (HmiRQP0315) were obtained from FulenGen (Guangzhou, China). The other primer sequences were as follows: MTDH: 5′-GAC​CTA​GCC​CAG​CTG​AAG​AAT-3′ and 5′-TTT​GCA​GTT​ATA​CTT​CGG​GGA-3’; GAPDH: 5′-ATG​GGT​GTG​AAC​CAC​GAG​A-3′ and 5′-CAG​GGA​TGA​TGT​TCT​GGG​CA-3′; and U6: 5′-GAC​AGA​TTC​GGT​CTG​TGG​CAC-3′ and 5′-GAT​TAC​CCG​TCG​GCC​ATC​GAT​C-3′. The relative gene expression was determined by the 2^–ΔΔCT^ method.

### Western Blotting Analysis

The cells were lysed with RIPA buffer. The antibodies against MTDH, occludin, claudin-1, NF-κB/p65, p-NF-κB/p65 (Ser536), Wnt3a, Axin-1, β-catenin, β-actin, and GAPDH were obtained from CST, Inc. (Cambridge, United States). The ImageJ software was used to quantitate the protein expression.

### Target Prediction and Luciferase Reporter Assay

The UCSC Genome Browser (http://genome.ucsc.edu/) was used to predict p65 binding sites (Gonzalez et al., 2021). miRNA targets were predicted using TargetScan (http://www.targetscan.org/) and miRWalk (http://zmf.umm.uni-heidelberg.de/apps/zmf/mirwalk2/).

The 3′-untranslated region (UTR) fragment of predicted genes was synthesized by Sangon (China) and subcloned into a pGL3 vector. The Promega dual-luciferase reporter assay was performed as previously reported (Mankertz et al., 2009).

### Chromatin Immunoprecipitation

ChIP-IT Express Enzymatic Kit (Active Motif) was used in this study. IPed DNA was assessed by qPCR. The sequences of the primers were as follows: MIR21: 5′-GAG​CCA​CTA​CCA​AGG​CAT​GT-3′ and 5′-CCA​ATC​CCA​GAG​GTG​CCA​TT-3’.

### Wnt Reporter Assay

The Wnt pathway–specific firefly reporter array (SA Biosciences) was performed in Caco-2 cells, and the luciferase assay was performed as described above.

### Statistical Analysis

GraphPad 8 software (California, United States) was used in data analysis. Spearman rank correlation was introduced to analyze the correlation between miR-21-3p and MTDH. All the experiments were performed in triplicate. *p* < 0.05 indicated significant differences.

## Results

### miR-21-3p Is Overexpressed in TNF-α–Induced Caco-2 Cells

**FIGURE 1:**
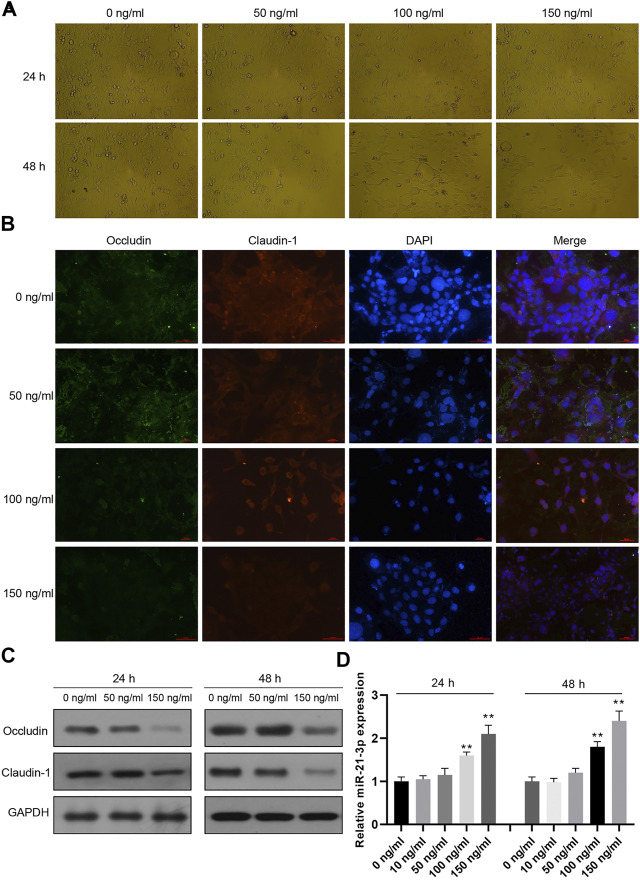
miR-21-3p is overexpressed in TNF-α–induced Caco-2 cells. **(A)** Caco-2 cells were treated with TNF-α for 24 or 48 h, subsequently followed by cell morphology using an optical microscope. The cell number was decreased, and the cell structures were evidently changed. **(B)** Caco-2 cells were treated with TNF-α for 24 h and immunofluorescent stained with occludin- or claudin-1–specific antibody and DAPI. **(C)** The expression of occludin and claudin-1 was evaluated by western blot analysis, showing that occludin and claudin-1 proteins were gradually decreased with the increasing concentration of TNF-α. **(D)** The expression of miR-21-3p was upregulated in a dose-dependent manner by RT-qPCR analysis. **p < 0.01, compared with the 0 ng/ml group.

Caco-2 cells were stimulated with different doses of TNF-α for 24 or 48 h, followed by cell morphology detection using an optical microscope. The results showed that the cell number was decreased and the cell structures were evidently changed, such as the appearance of vacuoles (Figure 1A). Moreover, occludin and claudin-1 proteins were decreased gradually with the increasing concentration of TNF-α (Figures 1B,C). In our previous study, miR-21-3p was reported to participate in intestinal barrier dysfunction. We subsequently detected varied miR-21-3p expression levels in Caco-2 cells. It was illustrated that the miR-21-3p expression level remained at a relatively low level when the TNF-α concentration was less than 50 ng/ml. As shown in Figure 1D, when the concentration was 100 or 150 ng/ml, miR-21-3p was dramatically overexpressed in a dose-dependent manner.

### miR-21-3p Is Upregulated by TNF-α Through NF-κB Signaling Pathway

**FIGURE 2:**
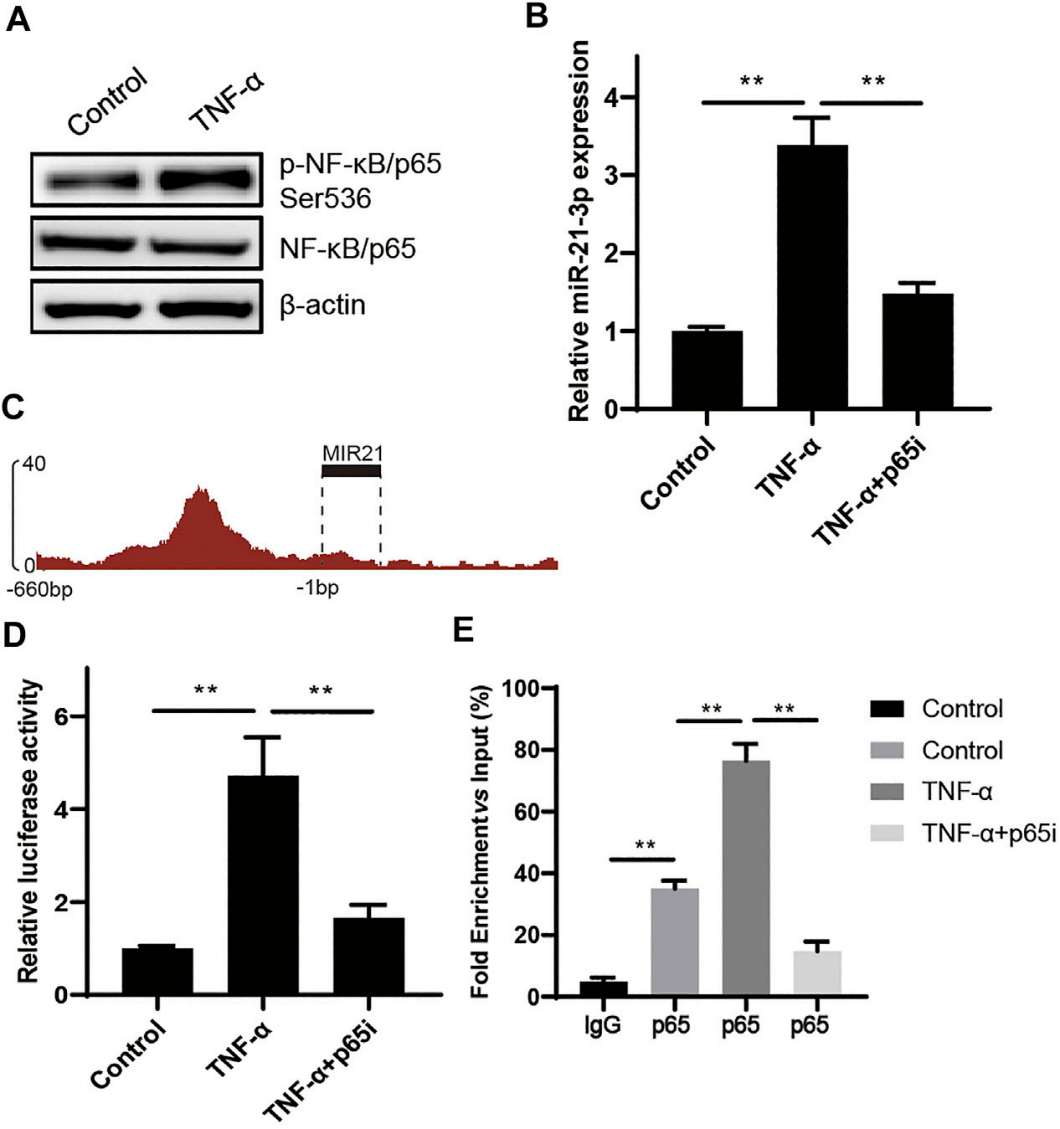
miR-21-3p is upregulated by TNF-α through the NF-κB signaling pathway. **(A)** The expression of p65 and p-p65 was detected in TNF-α–induced Caco-2 cells by western blot analysis. p-p65 was drastically increased in the stimulation group compared with the control. **(B)** The expression of miR-21-3p was evaluated in TNF-α–induced Caco-2 cells or TNF-α and parthenolide co-induced Caco-2. The overexpressed miR-21-3p induced by TNF-α was reversed by parthenolide. **(C)** Schematic representation of predicted p65 binding sites in the promoter region of MIR21. **(D)** Results of luciferase reporter assays in Caco-2 cells transfected with the reporter plasmid containing the MIR21 promotor region (−1–−660 bp). The activity of MIR21 promoter was promoted by TNF-α, which could be reversed by parthenolide. **(E)** Chromatin immunoprecipitation was performed using antibodies against p65, or a control IgG. Precipitated DNA was analyzed by qPCR using primers for MIR21. The graph shows the enrichment in DNA immunoprecipitated by the IgG or anti-p65 antibody compared with the input. **p < 0.01.

To verify how TNF-α contributed to miR-21-3p overexpression, we focused on the NF-κB signaling pathway, which is regulated by TNF-α and participates in gene transcriptional regulation (Ning et al., 2021). As shown in Figure 2A, the phosphorylated p65 (p-p65) was drastically increased in the cell lysate of the stimulation group compared with the control. As p-p65 could translocate from the cytoplasm to the nucleus and act as a transcriptional factor, we hypothesized that p-p65 could bind to the transcriptional regulatory elements of MIR21, which codes miR-21-3p. To test the hypothesis, we treated Caco-2 cells using TNF-α and parthenolide (p65 inhibitor) simultaneously. The result showed that the overexpressed miR-21-3p induced by TNF-α was reversed by parthenolide (Figure 2B), suggesting the linkage function of p65 from TNF-α stimulation to miR-21-3p expression. Next, we found that p65 was significantly enriched in the promoter region of MIR21 in the USCS database (Figure 2C). To verify whether p-p65 could regulate miR-21-3p expression by directly binding to the MIR21 promoter region, we performed the reporter assay and ChIP assay. It was illustrated that the activity of MIR21 promoter was promoted by TNF-α, which could be reversed by parthenolide (Figure 2D), and p65 was enriched in the MIR21 promoter region (Figure 2E). Our findings demonstrated that miR-21-3p could be upregulated by the TNF-α/NF-κB signaling pathway.

### miR-21-3p Directly Targets MTDH mRNA

**FIGURE 3:**
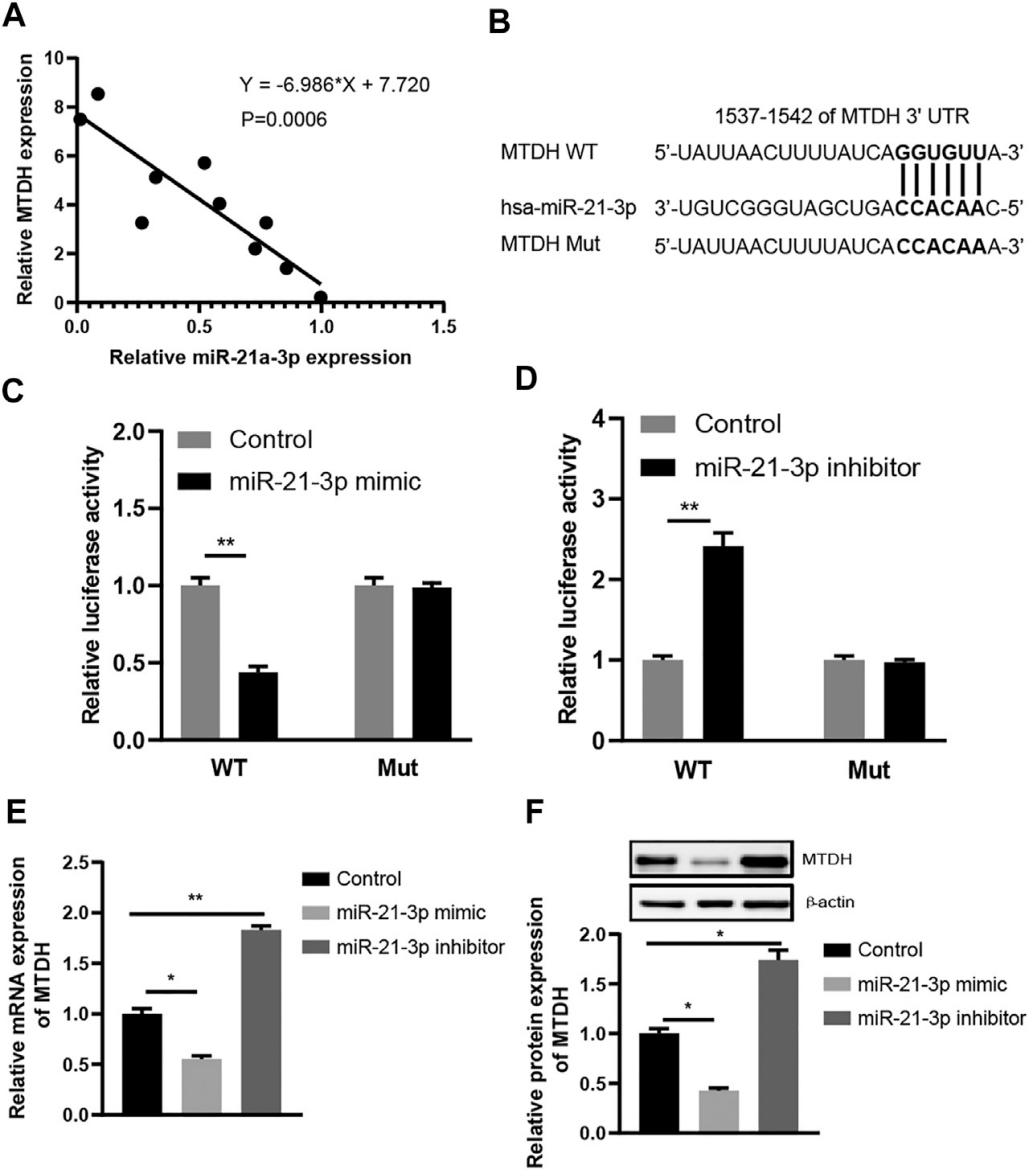
MTDH is a direct target gene of miR-21-3p. **(A)** Spearman rank correlation between miR-21-3p and MTDH levels in CRC tissues (*n* = 10) by RT-qPCR analysis. miR-21-3p expression was normalized to U6, and MTDH expression was normalized to GAPDH. The analysis results indicated that miR-21-3p expression was inversely related to MTDH. **(B)** Schematic representation of the predicted miR-21-3p binding sequence in the 3′-UTR of MTDH with wild-type form and with mutant form. **(C)** Results of luciferase reporter assays in Caco-2 cells, with co-transfection of WT or Mut 3′-UTR and miR-21-3p mimic, as indicated. **(D)** Results of luciferase reporter assays in Caco-2 cells, with co-transfection of WT or Mut 3′-UTR and miR-21-3p inhibitor, as indicated. **(E)** mRNA expression of MTDH in Caco-2 cells transfected with miR-21-3p mimic or inhibitor. Overexpression of miR-21-3p resulted in striking reduction of MTDH at the mRNA level. **(F)** Protein expression of MTDH in Caco-2 cells transfected with miR-21-3p mimic or inhibitor. Overexpression of miR-21-3p resulted in striking reduction of MTDH at the protein level. *p < 0.05, **p < 0.01.

microRNAs inhibit mRNA translation by directly binding to the mRNAs. We noticed that MTDH, a regulator of the Wnt signaling pathway, was predicted to be targeted by miR-21-3p. Due to the lack of the tissues from IBD patients, ten tissues from patients with CRC were enrolled. The analysis results indicated that miR-21-3p expression was inversely related to MTDH (r = −6.986, *p* = 0.0006, Figure 3A). We subsequently tested whether miR-21-3p could directly target MTDH mRNA to inhibit its translation (Figure 3B). We found that miR-21-3p markedly inhibited the luciferase activity in the group with wild-type MTDH-3′-UTR, but not in the mutant MTDH-3′-UTR group (Figures 3C,D). Furthermore, we also found that overexpression of miR-21-3p resulted in striking reduction of MTDH mRNA and protein (Figures 3E,F).

### miR-21-3p/MTDH/Wnt Signaling Pathway Is Involved in Intestinal Barrier Dysfunction

**FIGURE 4:**
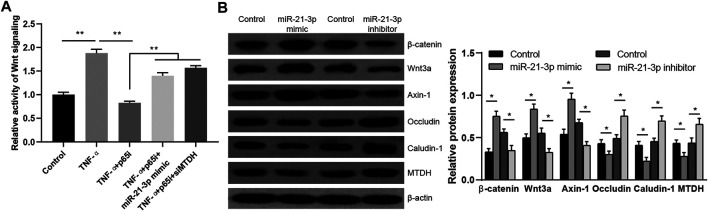
miR-21-3p/MTDH/Wnt signaling pathway is involved in the TNF-α–induced Caco-2 cell model. **(A)** The activity of Wnt signaling was evaluated by the Wnt reporter assay. The introduction of miR-21-3p mimic or downregulation of MTDH could reverse the inhibition by parthenolide. **(B)** The protein expression was detected in Caco-2 cells transfected with the miR-21-3p mimic or inhibitor. Wnt activation was evaluated with antibodies detecting the indicated Wnt pathway targets. *p < 0.05, **p < 0.01.

MTDH is reported to be a key modulator of the activity of Wnt signaling pathway (Shen et al., 2021a), and we hypothesized that TNF-α–induced miR-21-3p could regulate the activity of Wnt signaling pathway via MTDH. The Wnt activity reporter assay was performed. We observed a higher Wnt signaling activity in stimulated Caco-2 cells. Meanwhile, it was also observed that the introduction of parthenolide could significantly inhibit the Wnt signaling activity, and the introduction of miR-21-3p mimic or downregulation of MTDH could reverse the inhibitory effect of parthenolide (Figure 4A). Finally, we detected the protein expression of MTDH, claudin-1, occludin and Axin-1, Wnt3a, β-catenin in Caco-2 cells with upregulated or downregulated miR-21-3p. It was demonstrated that miR-21-3p reduced MTDH, claudin-1, and occludin expressions and induced Axin-1, Wnt3a, and β-catenin expressions (Figure 4B). These findings indicated that the miR-21-3p/MTDH/Wnt signaling pathway is involved in intestinal barrier dysfunction.

## Discussion

As one of the most important barriers of the body, normal intestinal epithelial tissue constitutes a physical and functional barrier structure. The intercellular junctions can regulate the absorption of nutrients and resist the invasion of toxins, antigens, and foreign microorganisms (Turner, 2006). Intestinal barrier dysfunction results in increased intestinal permeability to peritoneal endotoxin, thus promoting intestinal inflammation. Injury of intestinal barrier function is an important feature of many intestinal diseases (Salim and Soderholm, 2011; Halpern and Denning, 2015). Therefore, the impairment of intestinal barrier function seriously affects human health. Understanding the underlying mechanism of this phenomenon is important for the development of prevention and treatment of related diseases. As known, intestinal epithelial cells and their intercellular connective structures are crucial for the maintenance of intestinal barrier function (Al-Sadi et al., 2008). Multiple clinical studies have observed the upregulation of TNF-α in the intestinal tissues from patients with CD (Braegger et al., 1992; Murch et al., 1993; Reibetanz and Germer, 2015; Paredes et al., 2019). However, the exact mechanism by which TNF-α induces intestinal dysfunction remains unknown.

miR-21-3p was reported to be a motivator to promote intestinal barrier dysfunction, and its expression was correlated with the TNF-α level (Zhang et al., 2015; Zhang et al., 2018). To investigate the relationship between miR-21-3p and TNF-α, a series of experiments were performed. Our findings showed that phosphorylated p65 induced by TNF-α could promote the transcription of miR-21-3p *via* binding to the MIR21 promoter region.

Furthermore, our findings showed that MTDH, which is a type of scaffold protein, was regulated by miR-21-3p. MTDH is reported to participate in many key oncogenic pathways, including the Wnt signaling pathway by interacting with related proteins (Khan and Sarkar, 2021). The relationship between Wnt signaling and intestinal barrier dysfunction is complex and has never been truly determined. As a key regulator of epithelial proliferation, active Wnt signaling is essential to maintain epithelial homeostasis, and pathway inhibition results in crypt loss and tissue degeneration (Kuhnert et al., 2004). But the Wnt signaling pathway also could interact with multiple inflammatory signaling pathways (such as NF-κB signaling, MAPK signaling, AKT signaling, STAT signaling) to promote intestinal barrier dysfunction (Moparthi and Koch, 2019). In our study, we found that MTDH could reduce the expression of Axin-1, Wnt3a, and β-catenin and inhibit the activity of Wnt signaling pathway (Figure 4), which is contrary to the report by Kang (Shen et al., 2021b). Maybe, the modulation effect of MTDH on Wnt signaling pathway is dual-directed, by interacting with PRMT5, HSPA9, RPN1, HSPA5, AGPS, GTPBP1, ATAD3B, KIAA0020, or other proteins involved in the Wnt signaling pathway (Zhu et al., 2020).

## Conclusion

miR-21-3p mediates the changes in the Wnt signaling pathway through negative regulation of MTDH and plays a regulatory role in intestinal barrier dysfunction. Therefore, this study provides information on miR-21-3p as a potential biomarker and therapeutic target for intestinal barrier dysfunction and related bowel diseases.

## Data Availability

The original contributions presented in the study are included in the article/supplementary material, and further inquiries can be directed to the corresponding authors.
